# Effects of application of horticultural soil amendments on decomposition, quantity, stabilisation and quality of soil carbon

**DOI:** 10.1038/s41598-022-22451-2

**Published:** 2022-10-21

**Authors:** Sarah Duddigan, Liz J. Shaw, Paul D. Alexander, Chris D. Collins

**Affiliations:** 1 Department of Geography and Environmental Science, Soil Research Centre, University of Reading, Reading, RG6 6DW UK; 2 Department of Horticultural and Environmental Science, Royal Horticultural Society, Wisley, GU23 6QB UK; 3 Department of Product Development, Bulrush Horticulture Ltd., Co, Londonderry, BT45 8ND UK

**Keywords:** Carbon cycle, Biogeochemistry

## Abstract

Application of organic soil amendments is commonplace in horticulture to improve soil fertility. Whether this practice can also augment the soil carbon (C) pool has been of increasing interest in recent years. We used a controlled field experiment that has received annual applications of six different horticultural soil amendments for seven consecutive years. Each amendment was examined in terms of its contribution to bulk C and the distribution of C between theoretical pools, as defined by physical fractionation. Physical fractionation was combined with ^13^C nuclear magnetic resonance spectroscopy with cross-polarization and magic angle spinning (CPMAS NMR) analysis. Results indicated that the difference in total C concentration between treatments resulted from an increase in unprotected, free, particulate organic matter (fOM), rather than an increase in soil organic matter being occluded in aggregates or in organo-mineral complexes, and that C persisted in the fOM fraction as a result of accumulation in the alkyl C region. Unlike fresh litter or plant residues, organic amendments have undergone decomposition during the composting process (or during formation in the case of peat), in the absence of mineral soil components. This ex situ decomposition (and possible stabilization through acquired recalcitrance) could reduce the opportunity to become physically or chemically protected through association with the soil mineral phase following addition to soil. Carbon:Nitrogen (C:N) of amendment material likely influenced the rate of amendment decomposition. In addition, C:N determines the decomposition of plant litter inputs, as determined by the tea bag index.

## Introduction

Soil organic carbon (SOC) is commonly referred to as one of the most important indicators of soil quality^1^. It governs an array of soil physical, chemical and biological processes^2,3^ but its fate and behaviour are also influenced, in turn, by the physical, chemical and biological properties of the soil^4,5^. SOC, contained within the soil organic matter (SOM), holds approximately three times more carbon (C) than the atmosphere or terrestrial vegetation^6^, accounting for 80% of the terrestrial C pool^7^. Therefore maintenance of soil C stocks is critical, recognised during the 21st Conference of the Parties to the United Nations Framework Convention on Climate Change (COP21) in Paris, 2015. During this meeting, the ‘4 per mille Soils for Food Security and Climate’ action agenda was developed, as a voluntary action plan for partners in both the public and private sectors to increase agricultural soil C by 0.4%^8^.

There are two overriding factors which determine the bulk C content of a soil: the quality and quantity of the organic matter (OM) input; and the decomposition rate of this material^9^. SOM pools are often referred to as labile (active), intermediate (slow) or recalcitrant (passive/ stable) with turnover rates for these pools ranging from days to centuries depending on their stabilization mechanism^10^. There are three primary mechanisms for SOM stabilisation in soil^2,9,11–14^ which can be separated under the broad classifications of *biochemical* (intrinsic or acquired recalcitrance), *chemical* (sorption onto soil mineral components) and *physical* protection (occlusion within aggregates). Physical fractionation of SOM, particularly isolation of particulate organic matter and mineral associated organic matter, has been suggested as a valuable approach to better understand mechanisms of C stabilisation in soil^15^.

In the UK alone, horticultural production accounts for around 4% of the total area of cropland^16^, storing around 7.5 million tonnes C in the top 15 cm, based on the average C content of 43 t C ha^−1^ of agricultural and horticultural land in the United Kingdom (UK)^17^. In addition the potential for domestic gardens to store C is increasingly being recognised^18,19^ with an estimated 60–145 t C ha^−1^, being held in the top 100 cm of domestic garden soils^18,20^. The horticultural sector is distinct in the prevalence of use of organic ‘compost-type’ amendments. Application of organic amendments is commonplace in horticulture to improve potential for horticultural production through nutrient provision, improved soil structure and soil moisture retention as a result of increased SOM content. In addition, these materials could also theoretically add to the SOC sink.

The application of organic soil amendments in horticultural systems, such as manure and composts (i.e. green waste compost, spent mushroom compost etc.) has been reported to increase SOC^21–23^. Furthermore, application of mature composts, has been proposed to enrich hydrophobic compounds that protect labile soil C from mineralisation and increase the capacity of the SOC sink^24^. However knowledge of the fate of added C, and the mechanisms governing its long-term storage is limited.

An understanding of the mechanisms that stabilise SOC is necessary before recommendations for management of organic amendments in horticultural systems can be made^14,25,26^. However, little is known about the fate of organic amendments in soils in commercial and domestic horticultural systems. This is, in part, due to that fact the majority of understanding of C stabilization, and fate of input C, comes from systems that receive inputs of C that are dominated by fresh plant litter or crop residues (e.g. arable, grassland, forest). Although agricultural systems can also include decomposed organic amendments (e.g. anaerobic digestate and farmyard manure) horticultural amendments (e.g. garden compost, composted bark and peat) often differ from those commonly used in agricultural systems.

This study used samples from a controlled field experiment that received annual applications of six different horticultural soil amendments, such as spent mushroom compost and peat, for seven consecutive years. Each amendment was assessed in terms of its contribution to bulk SOC, and its effect on the distribution of SOC between theoretical pools, as defined by physical fractionation in both the topsoil and subsoil.

The aim of this research is to examine the effects of application of organic horticultural soil amendments on:

1. The amount of bulk C being applied to the soil, based on typical horticultural practise.
2. The total C content and the total C stock of amended soils.
3. The stabilisation mechanism of soil C in amended soils.
4. The composition of C stored in soil fractions, according to ^13^C nuclear magnetic resonance spectroscopy with cross-polarization and magic angle spinning (CPMAS NMR).
5. SOM decomposition, using the Tea Bag Index (TBI) decomposition rate as a proxy.

## Materials and methods

### Experimental site

This research took place on a controlled field experiment at the Royal Horticultural Society’s (RHS) Deer Farm in Wisley, U.K. (51.323428° N, -0.474392° W). The soil texture was a sandy loam and classified as a Luvisol according to the National Soil Resources Institute (NSRI) World Reference Base (WRB)^27^, accessed through the UK Soil Observatory (UKSO)^28^. Further details of the site can be found in Alexander et al.^29^. The initial soil organic matter content according to loss on ignition before the amendments were applied was 6.06% ± 0.16 (See Duddigan et al.^30^ for further initial analysis).

**Figure 1:**
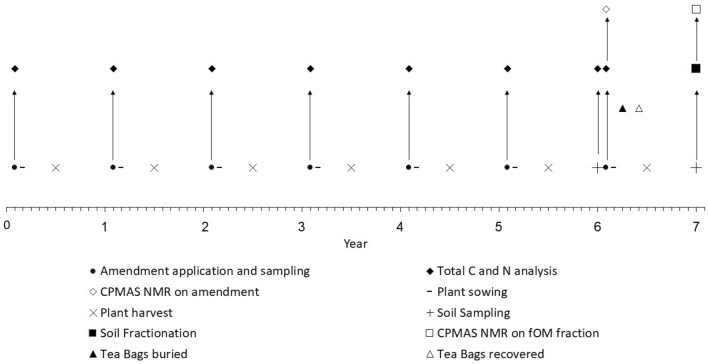
Timeline for sampling and analysis strategy. Plant selections: Year 0—Sunflower (*Helianthus annuus* ‘Antonil’); Year 1—Borage (*Borago officinalis*); Year 2—Garden cosmos (*Cosmos bipinnatus* ‘Sonata White’); Year 3—Nigella (*Nigella damascene* ‘Miss Jekyll’); Year 4—Honeywort (*Cerinthe major* ‘Purpurascens’); Year 5—Marigold (*Calendula officinalis* ‘Neon’); Year 6—Fiddleneck (*Phacelia tanacetifolia*); Year 7—Mallow (*Lavatera trimestris* ‘Ruby Regis’).

The site consisted of 3 × 3 m plots in a randomised complete block alignment (eight blocks, eight replicates) that had received annual applications for 7 years of either: Irish moss peat (Pt); composted horse manure (H), garden compost at full rate (GCf) and half rate (GCh) from collected prunings and cuttings from RHS Wisley Garden; composted bracken (Br) *Pteridium aquilinum* L. Kuhn blended with animal manure; composted bark (Bk); and spent mushroom compost (M), a by-product of the mushroom industry, which is a blend of wheat straw, gypsum and animal manure. Additional control treatments of (i) bare plot—no amendments applied, no plants grown (BP); and (ii) plants sown but no amendments applied (BP + P) were also used. Different horticultural plants were also grown annually from seed, to better represent the garden scenario (Fig. 1). Within horticulture, it is commonplace for amendments to be applied by volume and/or depth^29^. Therefore, the amendments were applied annually in early spring as a 5 cm layer on the surface of the soil (with the exception of the half rate garden compost treatment which received 2.5 cm) and incorporated into the top 15 cm of soil with the use of a rotovator.

Figure 1 details the timeline and sampling on the site, details of each component can be found in the sections to follow.

### Amendment sampling

All amendments had been purchased as commercial products annually, from the same suppliers. With the exception of the Wisley garden compost, which was generated on site using plant waste arisings from the RHS botanical garden in Wisley. The plant arisings were stockpiled, shredded and then windrow composted for 6–9 months before use (windrows turned monthly). Each year, three replicate subsamples were taken from each amendment. In the case where amendments were delivered in a bulk load, subsamples were taken from three different places within the pile. In the case where amendments were delivered in bags, subsamples were taken from three different bags.

### Soil sampling

Soil samples were taken from all plots using an auger at 0–15 cm and 15–30 cm depth. These depths were selected to account for the depth to which the amendments were incorporated in the soil by rotovator (0–15 cm) and the sub-soil below. Sampling below 30 cm was not possible on this site due to a thick layer of gravel at 30 cm. There were no obvious horizons aside from the boundary between the 0–15 cm and the 15–30 cm increments. Soils were sampled in Year 6, a year after the 6th application of amendments, just before the 7th application (Fig. 1). Auger samples were taken from three random positions within the central 2 × 2 m of the plot, to avoid possible boundary effects, and homogenised before taking a subsample for bulk soil C and nitrogen (N) analysis (see below).

Further soil samples were taken from three randomly selected blocks in Year 7, a year after the 7th application of amendments, just before the 8th application (Fig. 1) for SOM physical fractionation (described below).

### Physical fractionation

Five treatments were selected for full physical fractionation: (i) bare plot control, no amendments, no plants; (ii) control, no amendments with plants; (iii) garden compost full rate; (iv) garden compost half rate; and (v) spent mushroom compost. This work was intended to build on initial observations made two years previously where different treatments were selected (peat and composted horse manure^31^). This, in addition to time and resources available, meant it was not considered necessary to conduct full fractionation on all treatments.

SOM was split into five different fractions: (i) dissolved OM (DOM); ii) free, or particulate, OM (fOM); (iii) intra-macroaggregate OM (iMaOM); (iv) intra-microaggregate OM (imiOM); and (v) mineral associated OM (MinOM). These were obtained using the method described by Plaza et al.^32^. This method was selected both for its relatively low resource requirement, while still providing information on occlusion in two different aggregate size classes, free OM, and chemically protected OM. Briefly, a 20 g soil subsample (< 2 mm air dried), obtained with the use of a sample splitter was added to 80 ml sodium polytungstate (SPT), at a density of 1.85 g ml^−1^, shaken on an orbital shaker (1 revolution s^−1^) for 30 s and centrifuged at 2500 *g* for 30 min. The fOM, which was the floating light fraction, was separated through suction and filtration, washed thoroughly to remove residual SPT, and dried at 70 °C in a pre-weighed aluminium drying tray. The residual SPT from this step was set aside for subsequent steps, taking care not to dilute with water and reduce the density. The heavy fraction was transferred to a ‘microaggregate isolator’ (described by Six et al.^13^ with 50 glass beads (4 mm diameter) and shaken at 150 reps min^−1^ on a reciprocating shaker under a continuous, steady deionized water flow of about 0.2 L min^−1^. This breaks up macroaggregates into its components defined by two size classes: (i) > 250 µm which consisted of course particulate OM and sand; and (ii) < 250 µm which contained stable micro aggregates, fine particulate OM and sand, silt and clay. These were oven dried at 70 °C and recombined back into a single sample. The sample was then added to the SPT filtrate from earlier, shaken on an orbital shaker (1 revolution s^−1^) for 30 s, and centrifuged at 2500 *g* for 45 min. The iMaOM, which was the floating light fraction, was separated through suction and filtration, as described previously for fOM. The heavy fraction was recombined again with the SPT, dispersed by sonication (1500 J g^−1^) to break up the microaggregates, and centrifuged at 2500 *g* for 60 min. The light fraction (imiOM) and the heavy fraction (MinOM) were again separated by suction and filtration, washed thoroughly with deionised water and transferred to a pre-weighed aluminium drying tray. The SPT was retained, and dried for analysis of DOM. All drying trays were placed in a 70 °C forced air oven overnight and weighed. Mean C recovery of the physical fractionation was 98.2% ± 6.55.

Resources didn’t allow for all soil samples to be subjected to full physical fractionation. However, the fOM fraction was of particular interest as it appeared to account for the majority of the soil C in amended soils (see “Results and discussion”). Therefore, because the fOM is extracted in the first step of physical fractionation, samples from three plots of all amended treatments (Composted bark; composted bracken; garden compost; composted horse manure; spent mushroom compost; peat) were subjected the initial SPT flotation step of the physical fractionation method described above, and only the fOM was isolated.

### C and N content

Bulk oven dried whole soil, soil fractions, and amendment subsamples were ground to 0.2 mm using a disc mill and analysed for total C and N content on a Thermo Scientific Flash 2000 CN Analyser.

### Estimating C application rate

Using the mulch depth of 5 cm (with the exception of half rate garden compost which had a mulch depth of 2.5 cm), total C content, and the bulk density of the amendment, the total C applied per m^2^ of soil per year was estimated.

### Soil bulk density and estimating soil C stock

Dry soil bulk density at 0–15 cm was measured using a standard cylinder and driving tool method. This was done in the centre of each plot, immediately before soil sampling to avoid disturbance. Using the soil bulk density, total C content, and based on a depth of 15 cm, the soil C stock in top 15 cm was estimated (in kg C m^−2^) for each plot. This was calculated by multiplying the soil mass per unit area (bulk density x volume) for each plot by the C content.

### Tea bag index

Adopting a traditional litter bag approach with bags filled with amendments was not possible in this study due to the management of the site (rotovator use), causing disturbance to litter bags. Therefore, in order to quantify decomposition rate we used the Tea Bag Index (TBI), as described by Keuskamp et al.^33^, which uses tea bags as a standardised litter bag. Three bags of Lipton Green Tea (Unilever EAN:87 22700 0552 5) which is considered more labile (C:N ~ 12) and three bags of Lipton Rooibos Tea (Unilever EAN: 87 22700 18843 8) which is more recalcitrant (C:N ~ 60) were buried, per plot. Tea bags were buried in pairs (1 green, 1 rooibos) in three random locations in the central 2 × 2 m. Tea bags were buried to 8 cm depth in holes dug using a bucket auger and recovered after 90 days. Dried masses (70 °C) of the tea bags were recorded pre and post incubation on a four-place balance. All tea bags were from the same batch number. An average was calculated to produce a single mass loss for green and rooibos tea on each plot, these values were used to calculate a decomposition rate for each plot using the calculations detailed in Keuskamp et al.^33^.

### Nuclear magnetic resonance spectroscopy

Solid-state ^13^C CPMAS NMR analysis was conducted on the three replicates of the seven ground amendments as applied, in year 6. In addition, fOM fractions obtained from soils treated with the seven amendments in year 7 (Fig. 1) were also analysed using CPMAS NMR. Half rate garden compost was not included in the analysis as this amendment treatment differed only with respect to quantity of amendment.

CPMAS NMR used a 500 MHz Bruker Ultrashield with a pulse power of 67.6 kHz, using a spin rate of 10,000 Hz, with the magic angle set with an adamantane reference. The NMR was retuned between each sample, with a 500 µs contact time and a recycle delay of 2 s. More details on acquisition parameters can be found in the supplementary information (Table S1).

CPMAS NMR spectra were analysed based on the relative proportions of each of the following chemical shift regions: (i) alkyl C (0–50 ppm); (ii) O-alkyl C (50–110 ppm); (iii) aromatic C (110–160 ppm); and (iv) carbonyl C (160–200 ppm). The carbohydrate C/methoxyl C, or CC/MC, ratio (70–75 ppm/52–57 ppm), is described as a robust indicator of the level of decomposition of litters by Bonanomi et al.^34^. In addition, because the polymethyl C peak (30–35 ppm) appeared prevalent in the spectra obtained in this research, an additional carbohydrate C/polymethyl C, or CC/PMC, ratio was also calculated. It has been suggested that the rise in the proportion of alkyl C is a result of selective preservation of polymethylene over labile carbohydrate^35^ which is why the CC/PMC ratio was deemed appropriate. All integrations were performed on Bruker Topspin Version 3.6.

### Statistical analysis

Bulk soil C was analysed using analysis of variance (ANOVA) using treatment and sample depth as factors, combined with Tukeys post-hoc testing (*p* < 0.05). Total C in each of the physical fractions was also analysed in the same manner using treatment as a factor. In order to further examine the changes in composition of the fractions, C:N was analysed for each treatment using fraction as a factor.

When integrals and decomposition indices were obtained from CPMAS NMR spectra, ANOVA with interactions was conducted using amendment/fOM and treatment as factors with Tukey’s post-hoc testing (*p* < 0.05).

Prior to analysis, a Levene’s test for equal variance was conducted and data was transformed, if necessary, to satisfy the Levene’s test. ANOVA and Levene’s test were performed in Minitab Version 19.

Principal components analysis (PCA) was performed on integrals obtained in CPMAS NMR, with cluster analysis and analysis of similarity (ANOSIM) based on Euclidean distances. All multivariate analysis was conducted using Primer Version 6.

### Ethical approval

Experimental research and field studies on plants complied with relevant institutional, national, and international guidelines and legislation.

## Results and discussion

### C contents of horticultural amendments

**Figure 2:**
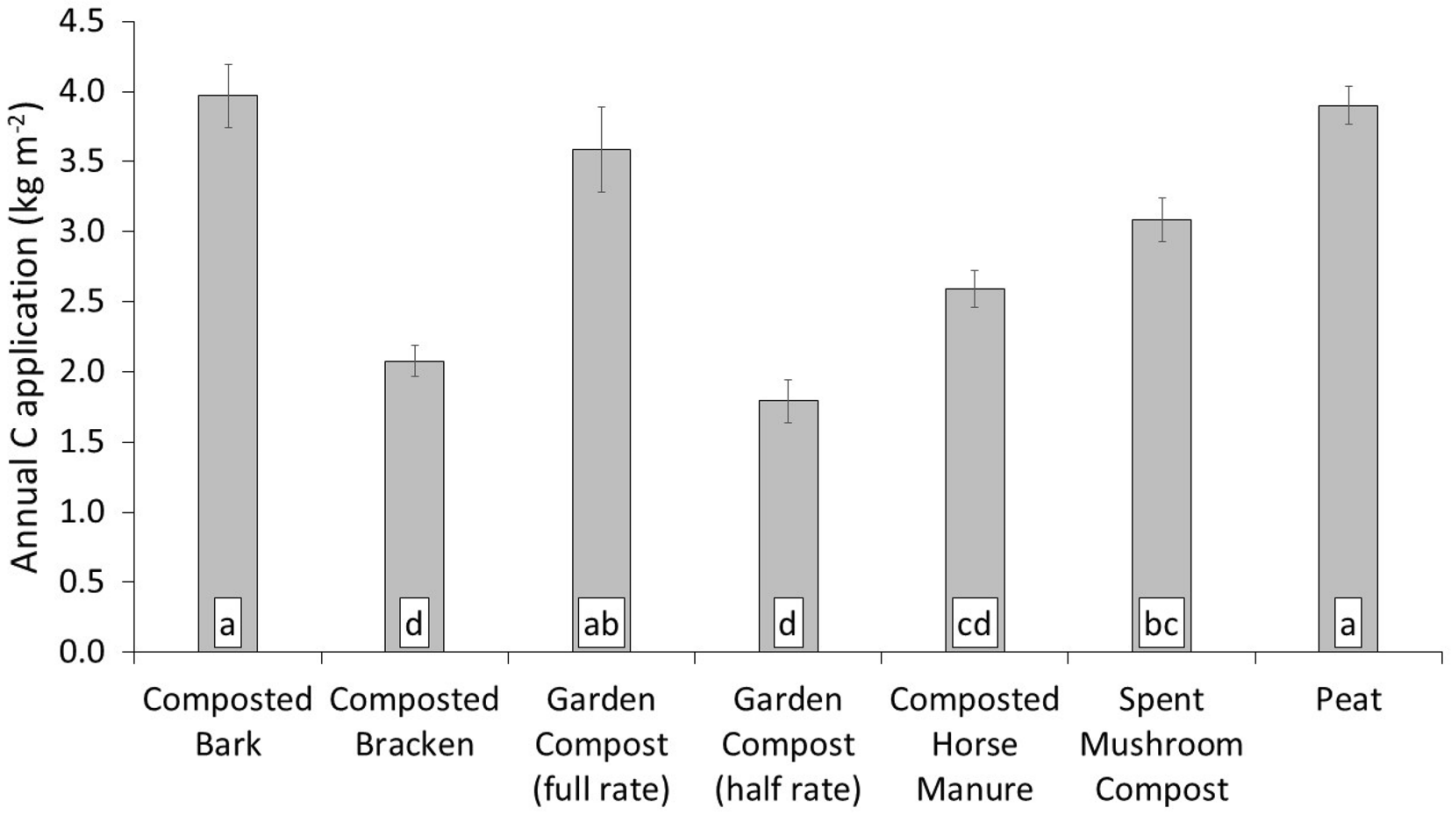
Annual C application of horticultural amendments. Mean of seven annual batches of amendments (n = 7). Error bars for standard error. Treatments that share a lower-case letter label have no significant difference according to one-way ANOVA and Tukeys post-hoc testing (*p* > 0.05).

The total C content of the amendments applied varied significantly (Table 1). This led to a significant difference in the quantity of C applied to each of the treatments (Fig. 2). With the highest total C percentage amendments, such as peat and composted bark, resulting in the highest mass of C applied to the soil. However, as application was determined by volume (5 cm thick mulch, or 2.5 cm in the case of the half rate), and the amendments differed in density, the garden compost (full rate), which had a comparably low percentage C content resulted in one of the highest total mass C applications. Application of a set thickness of mulch amendment, rather than a mass of C basis, simulated horticultural practice. Therefore, this will need to be considered in future management guidance in horticulture, if increasing C content of the soil is a desired outcome of application of soil amendments the thickness of the mulch will need to be adjusted depending on the material being applied.

**Table 1:** Dry density and proportional C content of amendments applied.

Treatment	Total C (%)	Total N (%)	C:N	Dry Density (g cm^−3^)*
Composted bark	45.5 ± 3.19b	0.9 ± 0.07d	54.5 ± 5.45a	0.18 ± 0.004bc
Composted bracken	41.0 ± 1.34b	1.9 ± 0.05ab	21.8 ± 1.14 cd	0.10 ± 0.008d
Garden compost	17.2 ± 1.51d	1.0 ± 0.08d	17.9 ± 0.97de	0.42 ± 0.022a
Composted horse manure	38.9 ± 0.99b	1.6 ± 0.15bc	25.7 ± 3.36c	0.13 ± 0.010cd
Spent mushroom compost	29.3 ± 1.42c	2.1 ± 0.06a	14.0 ± 0.61e	0.21 ± 0.012b
Peat	55.8 ± 1.45a	1.5 ± 0.05c	37.6 ± 1.42b	0.14 ± 0.005cd
*p*-value	< 0.001	< 0.001	< 0.001	< 0.001

Mean of seven annual (Year 0–6) batches of amendments (n = 7); ± standard error.

Treatments that share a subscript letter, for a particular variable, have no significant difference according to one-way ANOVA and Tukey’s post-hoc testing (*p* > 0.05).

*From Duddigan et al.^30^.

### Total soil C in amended soil

The influence of amendment was limited to the depth to which it was incorporated in the soil (0–15 cm), with no significant treatment effects in total soil C content, N content, or C:N observed deeper in the soil (15–30 cm, data not shown). Therefore, our discussion is limited to the 0–15 cm increment.

Application of all organic amendments resulted in a significantly higher C concentration than the unamended controls (Table 2), with the exception of the half rate compost having no significant difference to the control without plants. The highest observed C concentration was observed in the peat treatment, which also had among the highest C annual applications based on the 5 cm mulch (Fig. 2). However, peatlands are important for carbon sequestration in their own right, resulting in focussed attention on peatland conservation and reduction of the use of peat in horticulture^36^. Scottish peatlands, for example can contain 49% carbon^37^ and UK peatlands combined have been estimated to hold 3,000 million tonnes of carbon^38^. Therefore, any benefits of applying peat to horticultural soils, in terms of carbon sequestration, are drastically overshadowed by the losses of carbon in peatlands from which it is extracted. In addition, in terms of carbon addition, the same effects can be observed with a peat free alternative such as composted bark which had no significant difference in the C content of amended soil.

**Table 2:** Dry density and proportional C content of amended soil after six annual applications (Year 6).

Treatment	Total C (%)	Total N (%)	C:N	Bulk Density (g cm^−3^)
Control (no plants)	4.0 ± 0.39de	0.3 ± 0.01d	12.6 ± 1.08e	1.08 ± 0.027ab
Control (with plants)	3.4 ± 0.22e	0.3 ± 0.01d	12.5 ± 0.42e	1.14 ± 0.029a
Composted bark	13.7 ± 0.71a	0.5 ± 0.02c	29.5 ± 0.52a	0.50 ± 0.023d
Composted bracken	9.8 ± 0.86b	0.6 ± 0.04ab	16.1 ± 0.46c	0.50 ± 0.018d
Garden compost (full rate)	8.2 ± 0.34bc	0.5 ± 0.02abc	15.4 ± 0.08 cd	0.78 ± 0.018c
Garden compost (half rate)	6.1 ± 0.28 cd	0.5 ± 0.02c	13.2 ± 0.24de	0.96 ± 0.038b
Composted horse manure	6.8 ± 0.60c	0.5 ± 0.03bc	13.5 ± 0.59de	0.82 ± 0.037c
Spent mushroom compost	8.4 ± 0.54bc	0.6 ± 0.03a	13.3 ± 0.24de	0.73 ± 0.029c
Peat	14.9 ± 0.92a	0.6 ± 0.03ab	25.6 ± 0.72b	0.47 ± 0.034d
*p*-value	< 0.001	< 0.001	< 0.001	< 0.001

Mean of eight replicate experimental plots in Year 6 (n = 8); ± standard error.

Treatments that share a subscript letter, for a particular variable, have no significant difference according to one-way ANOVA and Tukeys post-hoc testing (*p* > 0.05).

Application of garden composts, comprising of food waste, grass clippings, plant prunings, etc. have been suggested to have great potential to increase soil carbon contents^39^, our results on garden compost from plant pruning’s support this. However, the potential of these materials to mitigate climate change is dependent on the alternative disposal methods available, if not applied to land as compost. For example, some disposal methods of these materials have climate mitigation potential in their own right, such as anaerobic digestion generating biogas as an alternative to fossil fuel use^40^. That said, waste fibre from anaerobic digestion is also used in some commercial soil amendments^41^.

**Figure 3:**
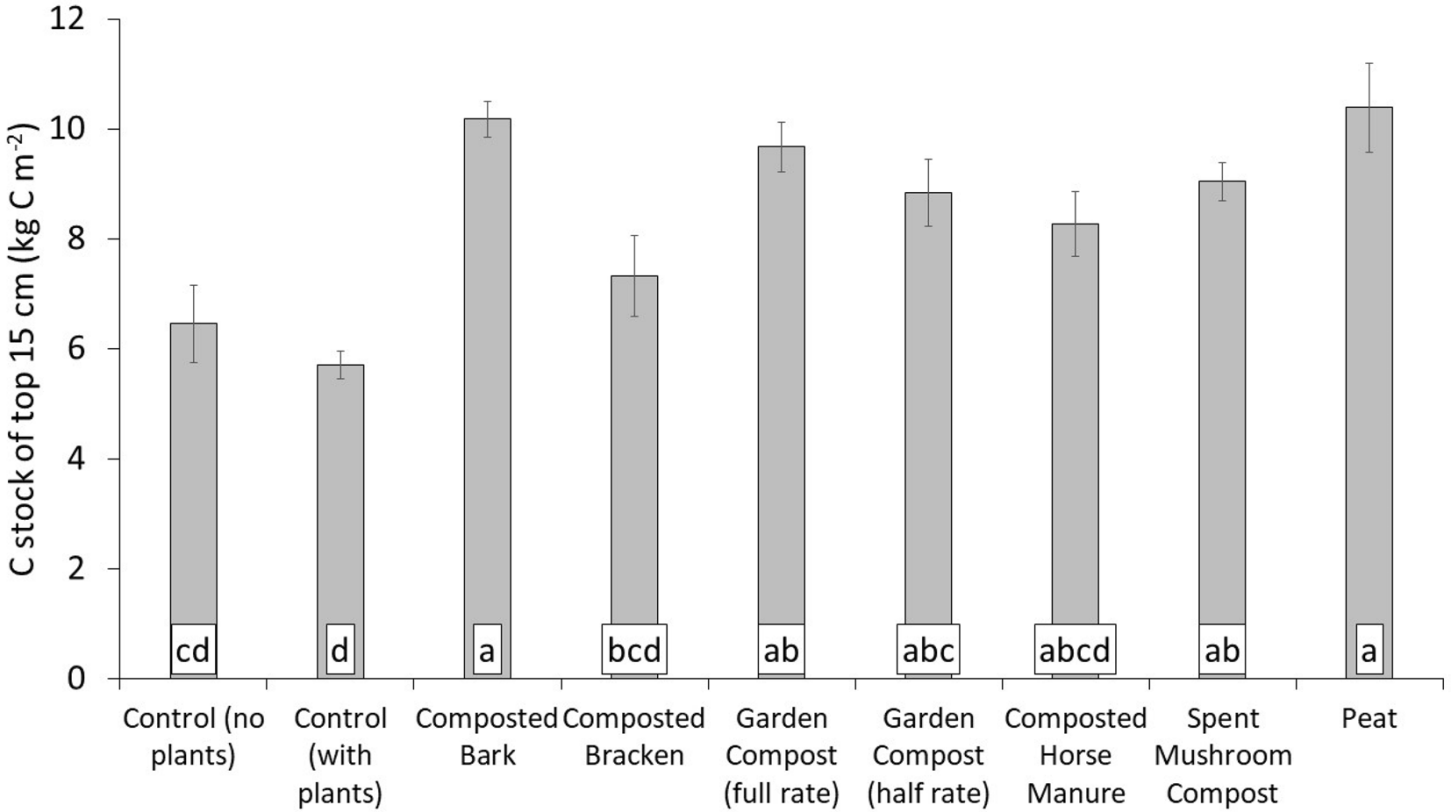
Soil C stock of the top 15 cm of amended soil after six annual applications (Year 6). Mean of eight replicate experimental plots in Year 6 (n = 8). Error bars for standard error. Treatments that share a lower-case letter label have no significant difference according to one-way ANOVA and Tukeys post-hoc testing (*p* > 0.05).

The total C stock of the top 0-15 cm of the composted bracken, composted horse manure and garden compost (half rate) was not significantly higher than the controls (Fig. 3). Despite the C content being affected by amendment application, the bulk density of the surface soil (Table 2) impacts the total C stock. This has implications for management and climate modelling, and how C contents should be reported in the literature. The Food and Agriculture Organization of the United Nations, for example, recommend that C stocks are considered (calculated as we did using the content, bulk density and depth) in the evaluation of land use change and management, rather than contents^42^.

### Physical organic matter fractions

There was no significant effect of amendment treatment on concentrations of physically or chemically protected C. This means that the difference in total C concentrations resulted from differences in unprotected C under each treatment, particularly the fOM fraction (Table 3). This is in concordance with the findings of a study that used the method described by Plaza et al.^32^ to fractionate amended soils from this site two years previously^31^. Application of vermicompost has also been found to increase particulate organic matter in horticultural soils^5^. However, conclusions relating to the relative sensitivity of SOC physical fractions to amendment can vary depending on the fractionation method used^31,43^. Although the *p*-value for ANOVA on C content of fOM according to treatment was < 0.05, post-hoc testing was unable to distinguish any treatments that were significantly different from one another. However, if all amended treatments (garden compost at two application rates and spent mushroom) are grouped together and both no amendment controls are also grouped, ANOVA and Tukeys post hoc testing shows that treatments with amendments applied are significantly higher in fOM than no amendment controls (Table 3).

**Table 3:** C Content of physical fractions of 0–15 cm soil.

Treatment	Mass C in fraction (mg C g^−1^ soil)				
	DOM (Unprotected)	fOM (Unprotected)	iMaOM (Weakly physically protected)	imiOM (Strongly physically protected)	MinOM (Chemically protected)
Control (no plants)	4.3 ± 0.4a	6.4 ± 2.4a	4.1 ± 2.3a	6.6 ± 1.7a	7.1 ± 2.1a
Control (with plants)	4.6 ± 0.3a	3.9 ± 0.6a	3.4 ± 0.2a	9.6 ± 2.8a	4.0 ± 0.4a
Garden compost (full rate)	3.1 ± 0.4a	62.5 ± 2.0a	15.2 ± 4.6a	6.4 ± 0.9a	3.3 ± 0.8a
Garden compost (half rate)	4.8 ± 0.7a	14.7 ± 10.3a	7.0 ± 3.1a	8.0 ± 4.3a	5.8 ± 1.2a
Spent mushroom compost	4.4 ± 0.7a	57.0 ± 27.5a	6.5 ± 1.0a	4.7 ± 1.0a	3.3 ± 0.4a
*p-*value	0.279	0.022	0.069	0.711	0.144
Amended*	4.1 ± 0.4A	44.8 ± 11.4A	9.6 ± 2.1A	6.4 ± 1.4A	4.1 ± 0.6A
No amendment**	4.4 ± 0.2A	5.1 ± 1.3B	3.7 ± 1.0A	8.1 ± 1.6A	5.6 ± 1.2A
*p-*value	0.547	0.024	0.055	0.441	0.249

Mean ± standard error (n = 3).

Fractions: *DOM* dissolved organic matter, *fOM* free organic matter, *iMaOM* intra-macroaggregate OM, *imiOM* intra-microaggregate OM, *MinOM* mineral associated OM.

Treatments that share suffix lowercase letters, in the same column, are not significantly different according to one-way ANOVA and Tukeys post hoc testing (*p* < 0.05) for that particular fraction. Suffix upper case letters signify significant differences if all amended (garden compost or spent mushroom compost) or no amendment (controls) treatments are grouped together.

*Mean of garden compost (full rate), garden compost (half rate) and spent mushroom compost (n = 9).

**Mean of control (with plants) and control (no plants) (n = 6).

Free OM often comprises a large proportion of total C and is one of the more sensitive C fractions to changes in management e.g. till vs no-till^2^. The chemistry of fOM within the soil often resembles that of the organic amendment, or plant litter, input to the soil. However, there is some evidence of partial decomposition and microbial by-products in fOM^13^. A fast turnover time suggests that this is a labile fraction of the SOM in soils, which potentially provides a large proportion of the nutrients required by plants, depending on the C:N and other nutrient ratios^2,7,21^.

He et al.^44^ found that the C:N was reduced in the heavy (mineral associated) fraction compared to the floating free light fraction and the C:N in aggregates decreases with aggregate size class. In addition, Plaza et al.^32^ reported that, for two agricultural soils, the C:N of the fractions decreased in the order DOM > fOM > iMaOM > imiOM > MinOM. This suggests that the contribution of undecayed plant tissues (which may have an initially high C:N) to the SOM in the fractions decreases and the proportion of microbial biomass-derived SOM (C:N ~ 8) increases in the transition from unprotected to physically protected to chemically protected OM. This trend, however, was not observed in the amended soils in our study. Except for the bare plot control (without plants), there are no significant differences in C:N of the fractions within any of the treatments (Table 4). This is possibly a result of the composting process, or during formation in the case of peat, whereby the amendments do not contain ‘undecayed’ plant tissues. Microbial by-products of decay will also be reduced as the labile components have been metabolised during the composting process^45^. This raises the question of whether the amendments that are being tested here, in some cases, have decomposed in the absence of opportunity to become physically or chemically protected through association with the soil mineral phase. This could have resulted in the total C variability between treatments being defined by differences in the fOM fraction, governed by biochemical mechanisms, rather than physical or chemical mechanisms. Labile components, such as carbohydrates, are also known to stimulate the formulation of stable aggregates^46^ and application of peat has been found to be ineffective at stimulating the formation of slaking-resistant aggregates^47^. Therefore aggregate formation, and subsequent physical protection of organic matter may have been reduced in our amended soils.

**Table 4:** C:N of physical fractions of 0–15 cm soil.

Treatment	C:N				
	Control (no plants)	Control (with plants)	Garden Compost (full rate)	Garden compost (half rate)	Spent mushroom compost
DOM (Unprotected)	5.0 ± 1.2c	48.6 ± 43.9a	58.9 ± 28.5a	63.8 ± 59.8a	52.5 ± 47.9a
fOM (Unprotected)	13.1 ± 0.7a	14.8 ± 0.3a	14.6 ± 0.5a	14.7 ± 0.9a	12.7 ± 0.1a
iMaOM (Weakly physically protected)	11.9 ± 0.5ab	11.9 ± 0.5a	13.5 ± 0.1a	13.4 ± 0.3a	12.4 ± 0.1a
imiOM (Strongly physically protected)	11.0 ± 0.8ab	8.8 ± 0.8a	9.7 ± 1.8a	11.4 ± 0.8a	9.4 ± 1.1a
MinOM (Chemically protected)	8.9 ± 0.6b	8.6 ± 0.6a	26.6 ± 10.0a	12.2 ± 0.6a	11.4 ± 1.9a
*p*-value	< 0.001	0.580	0.140	0.594	0.588

Mean ± standard error (n = 3).

Fractions: *DOM* dissolved organic matter, *fOM* free organic matter. *iMaOM* intra-macroaggregate OM, *imiOM* intra-microaggregate OM, *MinOM* mineral associated OM.

Fractions that share suffix lowercase letters, in the same column, are not significantly different according to one-way ANOVA and Tukey’s post hoc testing (p < 0.05).

Although association of SOM with mineral components is recognised as an important mechanism, it is recognised that the capacity for this as a C storage mechanism is potentially finite, determined primarily by the proportion and surface area of the minerals present^48,49^. Once all active sites are filled, the mineral component of the soil will become saturated^50^, or mineral-associated SOM will become more labile as bonds between SOM and mineral surfaces become weaker as the distance between mineral surfaces and OM increases^49,51^. There was no significant difference in MinOM between treatments in this investigation. However, based on the levels expected for saturated soil outlined by Hassink^52^, the soil has not yet reached saturation.

### Composition of C in amendments and the fOM fraction

**Figure 4:**
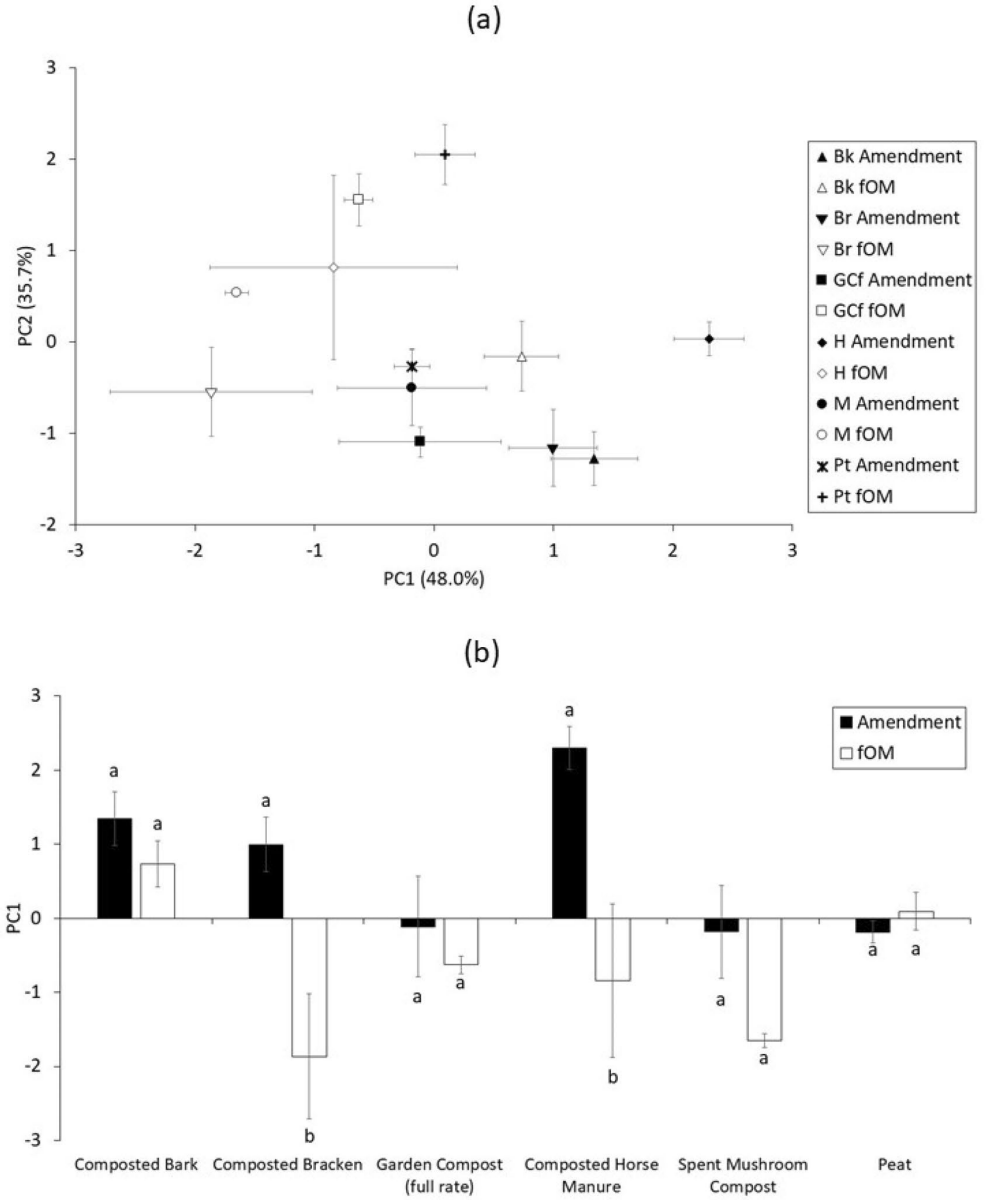
Principal components analysis of C integral functional groups in amendments applied and free organic matter (fOM) isolated a year later (**a**) Biplot of mean PC1 and PC2 for amendments and fOM (n = 3). Vectors for PC1: Alkyl C = − 0.52; O-Alkyl C = 0.71; Aromatic C = − 0.21; Carbonyl C = − 0.43. Vectors for PC2: Alkyl C = 0.58; O-Alkyl C = − 0.04; Aromatic C = − 0.70; Carbonyl C = − 0.41 (**b**) Scores for PC1 for each treatment. Bars within the same treatment that are labelled with the same lower case letter (a, b etc.) are not significantly different according to ANOVA and Tukey’s post hoc-testing (*p* > 0.05). Error bars for standard error (n = 3). Samples: *Bk* composted bark, *Br* composted bracken, *GCf* garden compost (full rate), *H* composted horse manure, *M* spent mushroom compost, *Pt* peat.

Due to the fact that fOM is not associated with mineral surfaces or aggregates, it is more likely that its stability in soils is determined by its biochemical recalcitrance^53^. Examining the compost-amended fOM and composted amendments first (i.e. excluding peat), the fOM of each treatment was represented by more negative PC1 scores when compared to the scores for the corresponding original amendment (Fig. 4), with the greatest relative change observed in composted horse manure. This is a result of an increase in the relative contribution of the alkyl C region and a decrease in O-alkyl C. An increase in AC/OAC ratio during decomposition has been observed in a number of studies due to the decomposition of labile carbohydrates and selective preservation of more recalcitrant waxes, in addition to subsequent build-up of secondary metabolites^34,54–56^. Alkyl C occurs in a number of structures, such as waxes and lipids, which require specific enzymes to decompose them^57^, and are therefore potentially more resistant to decay. It has also been suggested that the alkyl region is also indicative of the presence of microbial metabolites, that are produced during decomposition, further adding to this region’s accumulation in litter^58^.

During the compositing process, organic feedstocks are likely transformed to more biochemically stable materials via microbial humification reactions and mineralization of labile components^59^. It follows that a minimal change in composition of the corresponding fOM would imply a mature compost amendment that had undergone a significant degree of stabilisation during composting. Whereas an amendment comprising of partially decomposed or easily degradable material will stimulate increased soil microbial activity and respiration^60^. Therefore, it is possible that the composted horse manure was not as mature as the other composts. In addition to bringing about a reduction in labile components, the mineralization of organic carbon (to CO_2_) during composting also results in a decrease in the C:N with mature composts generally having a C:N below 20^61^. The C:N of 25 for the composted horse manure might thus further support that the composting process was not fully complete for this amendment. There was little change in relative composition between the composted bark amendment and the resultant fOM, according to the scores on PC1 (Fig. 3b), suggesting a lack of significant transformation since addition to soil. Given that a C:N of composted bark (~ 55) is far in excess of the critical C:N of ~ 25 (above which N limits decomposition^62^), transformation of composted bark in soil might have been N limited, but also potentially hindered by substantial lignification^63^, increasing biochemical recalcitrance. A relatively high C:N (~ 38) may also explain a lack of change in relative composition between the peat amendment and corresponding fOM, according to PC1 scores, although a separation between amendment and fOM across the PC2 axis is noted. Further analysis with ANOVA revealed that the proportional contribution of the aromatic C and carbonyl C did not differ significantly between treatments, or indeed within treatments for the amendment applied and the fOM (data not shown).

Although some observations have been made regarding the changes in composition of the amendments to fOM, analysis of similarity (ANOSIM) revealed that there was no significant difference between any of the samples, regardless of treatment, or whether it was an amendment or fOM (data not shown). This could, in part, be a result of the larger integral regions used. The O-alkyl region, for example is one of the more complex regions to analyse as a whole because it contains peaks that are attributed to some compounds traditionally thought of as labile (e.g. carbohydrate C), and others thought of as more recalcitrant (e.g. methoxyl C of lignin). Thus, some peaks in the alkyl C region will see a reduction and some will persist during decomposition. Therefore some, potentially more sensitive, decomposition indices were calculated (CC/MC and CC/PMC) using individual peaks, rather than larger integral regions.

**Figure 5:**
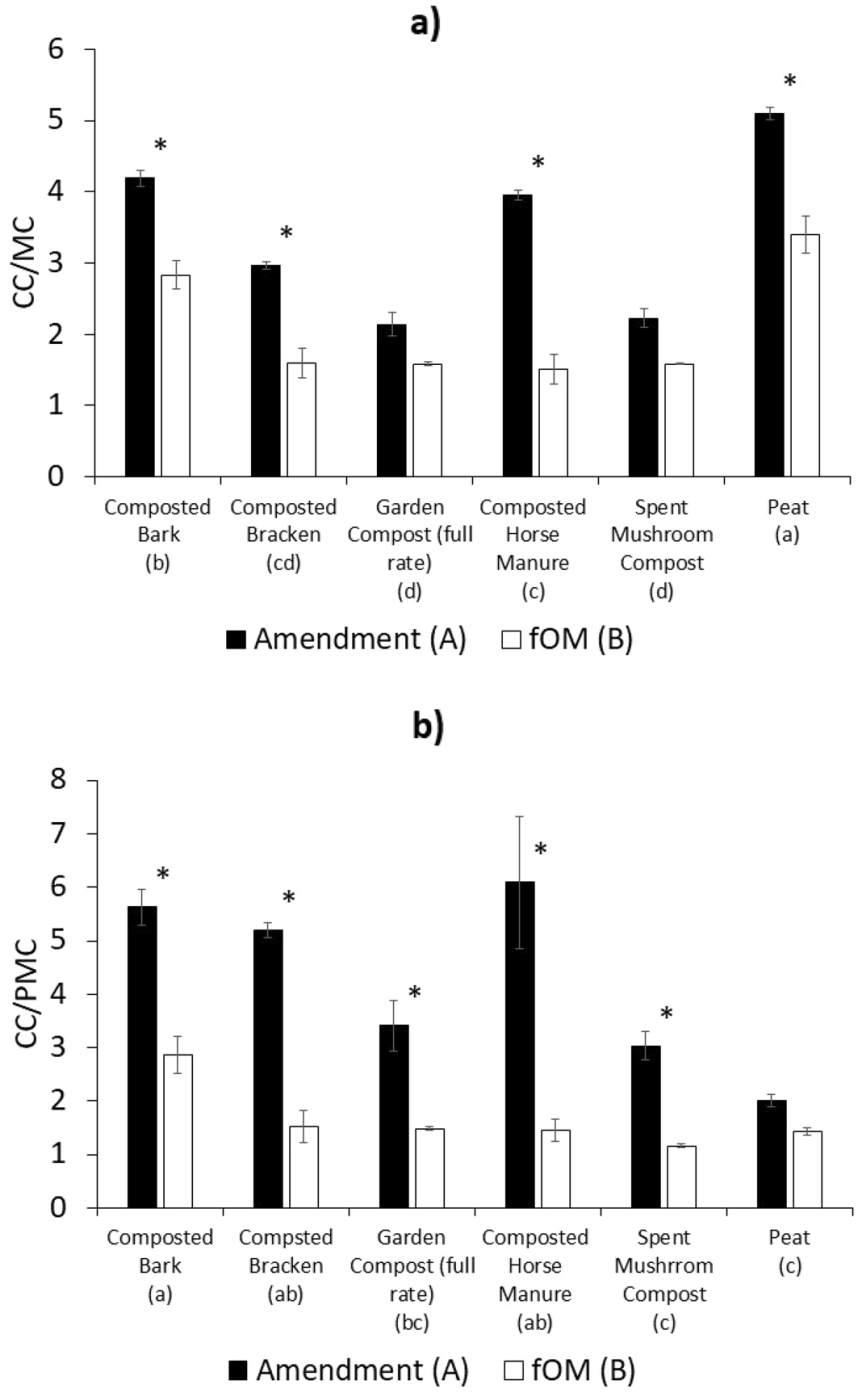
Decomposition indices of amendment applied and free organic matter in the soil the following year (**a**) carbohydrate C/methoxyl C ratio based on NMR integrals of 70–75 ppm/52–57 ppm; (**b**) carbohydrate C/polymethyl C ratio based on NMR integrals of 70–75 ppm/30–35 ppm. Error bars are standard error (n = 3). Samples that share the same suffix letters in brackets (lowercase for treatment, upper case for fOM/amendment) are not significantly different according to two-way ANOVA and Tukeys post hoc testing (*p* > 0.05). Treatments marked with a *signify those that have a significant difference between the amendment applied and the resultant fOM, for that treatment according to two-way ANOVA and Tukeys post hoc testing (*p* > 0.05).

There was no significant change in the CC/MC ratio observed in the spent mushroom compost, and the garden compost treatment between fOM and the amendment applied (Fig. 5a). Demethoxylation is often seen in the initial phase of decomposition of lignin^64^ and it is thought that only a limited group of fungi (white-rot fungi) are capable of mineralising lignin^65^. Unfortunately, analysis of the microbial community assemblage of the treated soils was not within the scope of this study, this information will be vital in future research into these amendments. In addition, there was no significant difference in CC/PMC in peat amendment applied and fOM (Fig. 5b). The CC/PMC ratio does however change significantly for the spent mushroom compost and garden compost, unlike the CC/MC ratio. This highlights the need to conduct multiple analyses when discussing decomposition.

On the whole, there was a relative decrease in C:N in the fOM compared to the amendment that was applied (Table 5), this is in keeping with a number of litter decomposition studies^34,55,66^. The spent mushroom compost treatment had the smallest relative change in C:N between the amendment applied and the fOM. The spent mushroom compost amendment also had the lowest initial C:N (Table 1) meaning that further decomposition in soil would not be N-limited with amendment C and N being immobilized in to microbial biomass with concomitant C and N losses as CO_2_ and NH_4_^+^.

**Table 5:** C:N of soil fOM isolated after 7 annual applications of amendments in relation to C:N of the amendments applied.

Treatment	Free organic matter C:N	Relative change in C:N compared to amendment before application (Table 1)
Composted bark	27.4 ± 2.7	− 0.497
Composted bracken	17.3 ± 1.8	− 0.206
Garden compost (full rate)	14.8 ± 0.2	− 0.173
Composted horse manure	15.6 ± 2.1	− 0.393
Spent mushroom compost	13.4 ± 0.1	− 0.043
Peat	27.0 ± 1.0	− 0.281

In contrast, the composted bark amendment (which had the highest C:N) saw the greatest relative decrease in C:N between the amendment and the resultant fOM. Although the NMR analysis suggested no substantial change in the chemical environment of the constituent C (discussed above), the bulk elemental analysis indicated that this amendment did undergo transformation on addition to the soil. Given the C:N, the N needs of microbial decomposers would not be satisfied by the N content of the amendment alone^62^. In consequence, it is likely that the microbial biomass mined the available soil N pool for metabolism, resulting in mineralization of C to CO_2,_ whilst retaining N within the decomposing biomass. This would lead to the amendment C:N to approach the C:N of the biomass that is decomposing it. This means that the decomposition dynamics of the amendment will be dictated by the C:N of the decomposers^67^.

Bonanomi et al.^34^ suggests that C:N only describes litter quality for undecomposed material and that CPMAS NMR was more successful at describing litter quality and predicting how it would break down in soils. The data presented here, however, suggest that the C:N of the material may still determine how an amendment is degraded in soil. C and N cycling within soil is inherently linked and the degree to which soil amendments influence these cycles will depend on the quality of the amendment, particularly the maturity of the amendment^68^.

The nature of CPMAS NMR means that the spectra obtained are not quantitative. Therefore any observations of increase or decrease of any organic components are related to the relative proportions of these compounds and are not quantitatively important observations. Secondly, in comparing the composition of the amendments applied on the 7th occasion with the composition of the fOM from the treated soil almost a year later, it is noted that the fOM analysed here was likely an integration of material accumulated over 7 years of organic amendment application**.** This situation differs from decomposition studies that use litter bags and retrieve litter for characterization after a fixed incubation time; the fOM being isolated and analysed here likely represented material that has experienced a range of incubation (decomposition) times. Whilst potentially desirable, adopting a litter bag approach to allow the characterization of chemical changes in the amendments with respect to incubation time was not possible in this study due to the management of the site, causing disturbance to litter bags. We thus consider the fOM characterization approach the best alternative option.

### Tea bag decomposition rate

The TBI uses a standardised material, of the same chemical quality and the same contact with the soil (i.e. in bags). However, the green and rooibos tea litter of the TBI did not resemble the amendments applied in our study in terms of (bio) chemical composition or their interaction with the soil (being buried in bags rather than surface-applied and incorporated). Therefore, the TBI did not provide information on how the amendments themselves were decomposing in this horticultural system. However, the TBI investigation does indicate how repeat application of amendments affects the decompositional environment of the soil.

**Figure 6:**
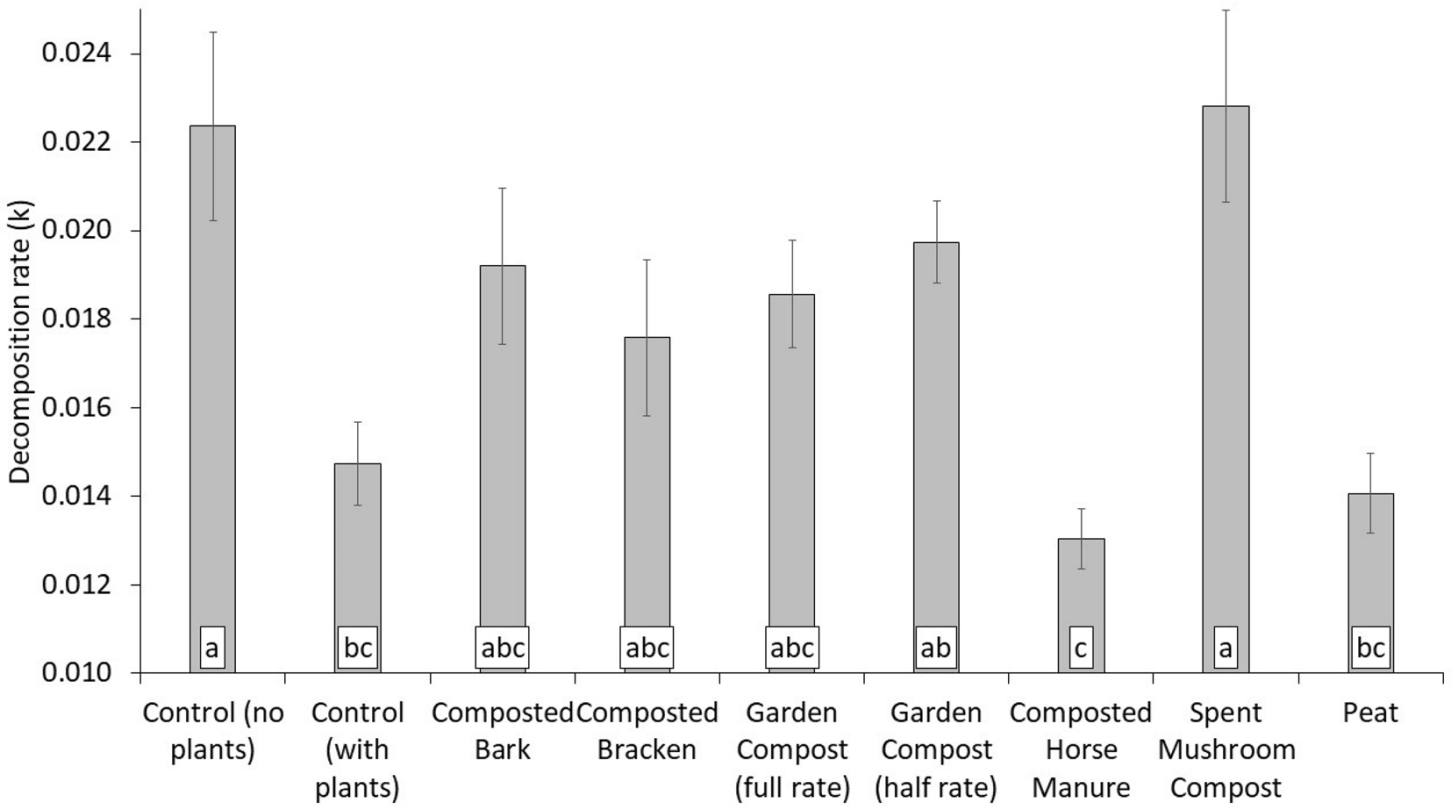
Decomposition rate under horticultural amendments according to the Tea Bag Index. Mean of eight replicate experimental plots in Year 6 (n = 8). Error bars for standard error. Treatments that share a lower-case letter label have no significant difference according to one-way ANOVA and Tukeys post-hoc testing (*p* > 0.05).

The highest decomposition rates were observed in the control treatment (without plants) and the spent mushroom compost (Fig. 6). This could be attributed to nitrogen availability to decomposers. Due to the high C:N (~ 60) of the rooibos tea^33^, decomposing microbes will have drawn in N from the surrounding environment in order to decompose this material^69^. Therefore decomposition rate was highest in the spent mushroom compost, the amendment which has the highest total (Table 1) and available^30^ nitrogen of the amendments applied. Composted horse manure and peat treatments, which had among the lowest decomposition rates, also had among the highest C:N (Table 1). Both composted horse manure and peat were above the optimum C:N of 25 for decomposable material according to Wang et al.^62^. High acidity in the peat treatment, for example, could also slow decomposition^45,70^. Although the control (without plants) treatment had less available nitrogen than amended treatments, it also did not have plants present to compete with decomposers for the nitrogen needed during decomposition. The presence of plants has been previously observed to reduce the decomposition rate, as determined by the TBI^71^.

## Conclusions

All of the amendments investigated in this research are commonly referred to under the blanket term of ‘compost’ in commercial horticulture products. However, we have demonstrated here that there was a significant difference between the amendments in terms of resultant soil carbon contents. This will have implications for practitioners, particularly if amendments are applied on a volume/depth basis. In physico-chemical terms, soil C can be unprotected, physically protected or chemically protected, with low medium and high residence times respectively in these pools. In the amended horticultural soils of this study, the most important mechanism for stabilisation of C in horticultural soil appeared to be biochemical recalcitrance of the, physico-chemically unprotected, fOM rather than mechanisms associated with adsorption onto mineral components or occlusion within aggregates.

On the whole, the traditional view of OM decomposition in soils, including a loss of labile carbohydrate and an accumulation of recalcitrant aromatic and alkyl C appears to hold true. However, this trend was not always statistically significant. This is likely due to the fact that, unlike many decomposition studies, the C input to these plots is not from fresh litter. All of the amendments applied to these plots have undergone some degree of humification, in the absence of mineral components, during the composting process (or during formation in the case of peat). Therefore, the amendment may have had opportunity to acquire a greater degree of biochemical recalcitrance than would normally make up fOM derived from fresh plant litter. Therefore, if managers move from application of composted amendments to application of more fresh material, for example, latent influences of treatment may differ. However, it is uncertain whether, with time, that physical occlusion or organo-mineral complexing may become more important to C stabilisation in amended soils the future. The C:N of amendment material, whilst likely influencing the rate of amendment decomposition, also determines the soil decompositional environment for plant litter inputs.

## Supplementary Information

Supplementary Information.

## Data Availability

Data available on request from the corresponding author.
