# Supplementary material for: Effects of application of horticultural soil amendments on decomposition, quantity, stabilisation and quality of soil carbon
Source: Sci Rep. 2022 Oct 21;12:17631. doi: 10.1038/s41598-022-22451-2 (PMC9586950; doi:10.1038/s41598-022-22451-2)

# Supplementary information

## Table S1 - Acquisition of CPMAS NMR spectra

*Amendments and fOM*

Spectral width = 401.6 ppm

Acquisition time = 0.041 sec

Receiver gain = 128

Dwell time = 9.900 µsec

Pre-scan delay = 6.50 µsec

Size of FID = 4096

|  |  | **Number of Scans** | |
| --- | --- | --- | --- |
| **Treatment** | **Replicate** | **Amendment** | **Free particulate OM fraction** |
| Composted Bark | 1 | 32768 | 4096 |
|  | 2 | 32768 | 18432 |
|  | 3 | 2048 | 18432 |
| Composted Bracken | 1 | 32768 | 20480 |
|  | 2 | 4096 | 20480 |
|  | 3 | 16384 | 20480 |
| Garden Compost | 1 | 32768 | 18432 |
|  | 2 | 32768 | 18432 |
|  | 3 | 32768 | 18432 |
| Composted Horse Manure | 1 | 32768 | 4096 |
|  | 2 | 2048 | 4096 |
|  | 3 | 2048 | 20480 |
| Spent Mushroom Compost | 1 | 36864 | 41984 |
|  | 2 | 49152 | 44032 |
|  | 3 | 49152 | 44032 |
| Peat | 1 | 32768 | 4096 |
|  | 2 | 4096 | 4096 |
|  | 3 | 4096 | 4096 |

## Figure S1 - 13C CPMAS NMR Spectra for Amendments and soil ‘free’ organic matter


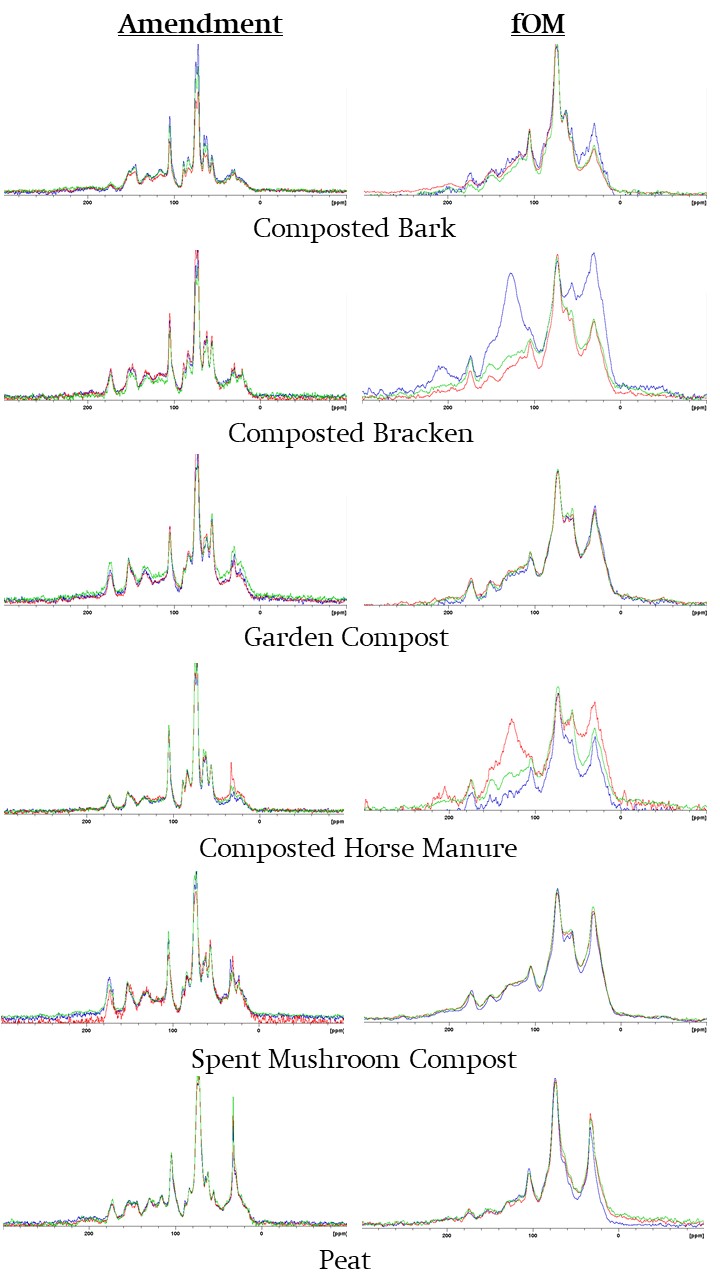

Supplement: Supplementary file 1 — Supplementary Information. [file 41598_2022_22451_MOESM1_ESM.docx]
